# Zoonotic *Yersinia enterocolitica* in Swine: One Health Implications for Food Safety and Public Health

**DOI:** 10.3390/vetsci12090795

**Published:** 2025-08-23

**Authors:** Sónia Saraiva, Juan García-Díez, Telma de Sousa, Rita Calouro, Diana Fernandes, Ana V. Mourão, Cristina Saraiva, João R. Mesquita, Ana C. Coelho, Patrícia Poeta

**Affiliations:** 1 Department of Veterinary Sciences, University of Trás-os-Montes and Alto Douro (UTAD), 5000-801 Vila Real, Portugal; crisarai@utad.pt (C.S.); accoelho@utad.pt (A.C.C.); ppoeta@utad.pt (P.P.); 2 Animal and Veterinary Research Center (CECAV), University of Trás-os-Montes and Alto Douro, 5000-801 Vila Real, Portugal; ritasousacalouro@gmail.com; 3 Associate Laboratory of Animal and Veterinary Sciences (AL4AnimalS), 5000-801 Vila Real, Portugal; 4 Department of Genetics and Biotechnology, University of Trás-os-Montes and Alto Douro, 5000-801 Vila Real, Portugal; telmaslsousa@hotmail.com; 5 Microbiology and Antibiotic Resistance Team (MicroART), University of Trás-os Montes and Alto Douro, 5000-801 Vila Real, Portugal; dianaivfernandes@gmail.com (D.F.); vanessamourao123@gmail.com (A.V.M.); 6 Functional Genomics and Proteomics Unit, University of Trás-os-Montes and Alto Douro, 5000-801 Vila Real, Portugal; 7 Associated Laboratory for Green Chemistry, University NOVA of Lisbon, 1099-085 Caparica, Portugal; 8 School of Medicine and Biomedical Sciences (ICBAS), Porto University, 4050-313 Porto, Portugal; jrmesquita@icbas.up.pt

**Keywords:** foodborne pathogen, risk factor, zoonosis, food safety, One Health, virulence gene, *Yersinia enterocolitica*

## Abstract

*Y. enterocolitica* is a cold-tolerant, foodborne pathogen that poses a significant risk to public health, with pigs identified as its main reservoir. This review explores the current knowledge on the epidemiology, transmission, and virulence of *Y. enterocolitica* in pigs, highlighting their central role in spreading the bacterium to humans—primarily through the consumption of raw or undercooked pork. The pathogen is frequently found in pig tonsils and intestines and can contaminate meat during slaughter. Among the various strains, bioserotype 4/O:3 is the most common cause of human illness. *Y. enterocolitica* carries a diverse set of virulence genes, such as *ail*, *yst*, *inv*, and *yad*, and evades immune responses. The review also covers major outbreaks, risk factors on farms and in slaughterhouses, and the limitations of current surveillance systems. Reducing the impact of *Y. enterocolitica* requires a One Health approach linking animal health, food safety, and public health.

## Simple Summary

This review aims to provide a comprehensive overview of *Yersinia enterocolitica* in pigs, with a specific focus on its transmission pathways and associated risk factors. *Y. enterocolitica* is a foodborne bacterium commonly found in pigs, which can spread to humans through contaminated pork. It carries several genes that help it cause disease and resist the immune system. The pathogen is difficult to control due to its ability to survive at low temperatures and its silent presence in healthy pigs. Improving farm hygiene, slaughterhouse practices, and surveillance is crucial to reducing its impact. A One Health approach that connects animal, food, and human health is essential. Future solutions may include vaccines or bacteriophage treatments to prevent infections more effectively.

## 1. Introduction

*Yersinia* are Gram-negative rods belonging to the *Yersiniaceae* family, which is part of the order of the Enterobacterales [1]. Yersiniae are oxidase-positive, gram-negative, facultative anaerobes that ferment glucose [2]. Within the *Yersinia* genus, the main pathogens for humans and animals are *Y. pestis*, the etiological agent of the plague (the black death), i.e., *Y. enterocolitica* and *Y. pseudotuberculosis*, both of which are responsible for enteric yersiniosis [3]. The consumption of contaminated pork products positions pigs as a significant source of human *Y. enterocolitica* infection [4,5]. Genetic studies have shown similarities between human and pig strains, supporting the role of pigs as an important source of human yersiniosis [6,7].

*Y. enterocolitica* is a significant zoonotic foodborne pathogen, with pigs being a major asymptomatic reservoir [8,9]. *Y. enterocolitica* can be transmitted from infected pigs to their carcasses during slaughter, potentially introducing the pathogen into the food chain [8]. Human infection is typically acquired through the consumption of contaminated pork, unpasteurized milk, and, in some cases, through exposure to contaminated vegetables or water sources [10,11,12]. *Y. enterocolitica* can also be found in companion animals, including dogs and cats, and wild animals such as rodents and game animals [13,14,15]. However, a study conducted in China found that pathogenic strains were predominant in pigs and dogs, whereas nonpathogenic strains were more commonly isolated from poultry and wildlife. The presence of shared pulsed-field gel electrophoresis (PFGE) patterns between animal isolates and human patients with diarrhea suggests that these animals may serve as a source of infection in the region [16].

*Y. enterocolitica* first emerged as a human pathogen in the 1930s [17]. The first confirmed *Y. enterocolitica* foodborne outbreak (FBO) caused by gastrointestinal illness occurred in the United States in 1976 in the rural community of Holland Patent, New York [18]. The source of infection was chocolate-flavored milk, which became contaminated with *Y. enterocolitica* after pasteurization [2]. Several FBOs caused by *Y. enterocolitica* infections during the 1980s brought greater visibility to the pathogen and stimulated further research into its epidemiology and pathogenicity. This pathogen, which is widely distributed in the environment and animals, can grow at low temperatures and in refrigerated foodstuffs [19]. The ability of *Yersinia* to survive and grow at low temperatures highlights the importance of thoroughly cooking food, especially pork, and maintaining proper hygiene during preparation to prevent yersiniosis [20]. *Y. enterocolitica* exhibits high genetic heterogeneity, as evidenced by its diverse biotypes, serotypes, and ribotypes [21]. In total, 6 biotypes (1A, 1B, 2, 3, 4, 5) and more than 70 serotypes of *Y. enterocolitica* have been identified [22]. The biotypes of *Y. enterocolitica* can be divided according to their pathogenic properties: non-pathogenic biotype 1A, weakly pathogenic biotypes 2–5, and the highly pathogenic biotype 1B [22]. Human pathogenic strains primarily belong to serogroups O:3, O:5,27, O:8, and O:9 [23]. The heterogeneity of *Y. enterocolitica* is also evident by the wide variation in its antimicrobial resistance profiles and PFGE genotyping results, showing diverse populations in both clinical and environmental samples [24]. Genetic diversity is further evidenced by the presence of various virulence genes, including *inv*, *ystA*, *ystB*, and *ail*, which contribute to pathogenicity [24]. Among these, the *ail* gene was for a long time exclusively associated with the pathogenic serotype, playing a crucial role in serum resistance and the invasion of host cells [25]. Moreover, the currently accepted and widely used method for distinguishing pathogenic from non-pathogenic *Yersinia* species primarily relies on detecting the *ail* gene (adhesion and invasion locus) [26]. However, virulence markers, including the *ail* gene, have also been identified in non-pathogenic *Yersinia* species as well as in *Y. enterocolitica* biotype 1A isolates [27]. The virulence of *Y. enterocolitica* pathogenic strains is associated with the presence of virulence genes and other markers that facilitate adhesion, invasion, and survival within the host. The EN ISO 10273 standard for detecting presumptive pathogenic *Y. enterocolitica* in foods was last updated in 2003, and its enrichment and plating steps have not undergone international collaborative validation [28,29]. Unlike traditional methods, the ISO/TS 18867 real-time polymerase chain reaction (PCR) technique has undergone rigorous validation, proving to be an effective and reliable alternative for the accurate identification of pathogenic *Y. enterocolitica* biovars [30]. A comprehensive literature search was conducted in PubMed, Science Direct, Embase, and Scopus to identify all published studies. The search strategy included the keywords “Yersinia enterocolitica,” “yersiniosis,” and related terms. Additionally, the bibliographies of the included articles were hand-searched to identify further relevant references. Studies were selected based on the following criteria: primary research articles (published or in press); investigations reporting the detection of zoonotic *Y. enterocolitica* in foods, pigs, or wild boar; clinical reports of human poisoning cases; studies conducted in a defined region or country; and availability of full-text articles. Studies were excluded if they did not specifically identify *Y. enterocolitica* species, biotypes, or serotypes.

## 2. Taxonomy and General Characteristics of Yersinia

*Y. enterocolitica* is part of the *Yersinia* genus, which includes both pathogenic and non-pathogenic species, and is most closely related to *Yersinia pseudotuberculosis* and *Yersinia pestis*. Its classification is based on a combination of biochemical properties, serotyping (O antigens), and molecular analyses such as 16S rRNA sequencing and multilocus sequence typing (MLST) [31,32]. Different species of Yersinia present varying pathogenic potential, including *Y. pestis*, the causative agent of plague; *Y. enterocolitica*, a major cause of foodborne gastroenteritis; and *Y. pseudotuberculosis*, which causes tuberculosis-like infections in animals and humans [1]. Other species, such as *Y. aldovae*, *Y. bercovieri*, *Y. intermedia*, *Y. kristensenii*, *Y. mollaretii*, *Y. rohdei*, *Y. ruckeri* (a fish pathogen), *Y. similis*, *Y. aleksiciae*, *Y. entomophaga*, *Y. wautersii*, and *Y. nurmii*, are primarily environmental or have limited pathogenicity. Yersinia can metabolize carbohydrates through both oxidative and fermentative pathways [33].

*Yersinia* are oxidase-negative, Gram-negative, rod-shaped bacteria belonging to the family Enterobacteriaceae. *Yersinia* species are distinguished by their small colony size and, under certain conditions, their cells may appear coccoid (spherical) [33]. *Y*. *enterocolitica* is characterized by psychrotrophic growth at temperatures as low as 4 °C, and it exhibits motility at 25 °C but not at 37 °C. It is also negative for phenylalanine deaminase and positive for urease activity [33].

*Y. enterocolitica* was initially divided into five biogroups (one to five) based on several biochemical reactions, namely, indole production, hydrolysis of aesculin and salicin, lactose oxidation, acid from xylose, sucrose, trehalose, sorbose and sorbitol, o-nitrophenyl-β-d-galactopyranoside, ornithine decarboxylase, Voges–Proskauer reaction, and nitrate reduction. Following modification of the scheme, there are now six recognized biotypes [33]. *Y. enterocolitica* biotypes 1B, 2, 3, 4, and 5 do not rapidly (within 24 h) hydrolyze esculin or ferment salicin (adapted from Wauters et al. [34]).

The biotyping scheme of *Y. enterocolitica* is nowadays based on a set of biochemical tests used to distinguish between six biovars: 1A, 1B, 2, 3, 4, and 5. Biovar 1A is positive for lipase, indole production, D-xylose fermentation, the Voges–Proskauer reaction, trehalose fermentation, nitrate reduction, pyrazinamidase, and β-D-glucosidase and shows variable reactions for esculin hydrolysis and proline peptidase. Biovar 1B is positive for lipase, indole production, D-xylose fermentation, the Voges–Proskauer reaction, trehalose fermentation, and nitrate reduction but negative for esculin hydrolysis, pyrazinamidase, β-D-glucosidase, and proline peptidase. Biovar 2 shows a delayed positive reaction for indole production and is positive for the D-xylose fermentation, Voges–Proskauer reaction, trehalose fermentation, and nitrate reduction. It is negative for lipase, esculin hydrolysis, pyrazinamidase, β-D-glucosidase, and proline peptidase. Biovar 3 and 4 are positive for the Voges–Proskauer reaction, trehalose fermentation, and nitrate reduction. Biovar 3 is also positive for D-xylose fermentation. Biovar 5 shows delayed positive reactions for Voges–Proskauer test and variable reactions for D-xylose fermentation. It is negative for all other tests (adapted from Wauters et al. [34]). Recent studies characterizing the genomes of prophages from pathogenic *Y. enterocolitica* have revealed two distinct, highly conserved clusters corresponding to serotypes O:3 and O:9, highlighting the role of tail fiber proteins in determining host specificity. These findings not only deepen our understanding of phage-driven evolution in *Y. enterocolitica* but also point to potential applications in the specific detection of pathogenic strains [35].

## 3. Virulence Factors

As mentioned before, *Y. enterocolitica* comprises six biotypes of which five (1B, 2, 3, 4, and 5) are considered to be pathogenic [22,36]. *Y. enterocolitica* belonging to biotype 1A are thought to be avirulent, based on the absence of the majority of virulence markers present in pathogenic strains. However, there is increasing evidence that at least some biotype 1A strains are associated with yersiniosis in humans [37]. Pathogenic *Y. enterocolitica* strains have traditionally been characterized by the presence of a 70-kilobase (kb) virulence plasmid (pYV), which encodes key virulence factors such as the adhesin Yersinia adhesion A protein (YadA) and the transcriptional regulator *virF*. Additionally, these strains carry several chromosomal virulence genes, including *invA* (invasin), *ail* (attachment and invasion locus), *ystA* (Yersinia stable toxin A), and *myfA* (mucoid Yersinia factor A) [38]. These virulence features, particularly those encoded by plasmids, drive *Y. enterocolitica* infection and enable the bacteria to persist within the human host [23]. The outer membrane of Gram-negative bacteria is primarily composed of lipopolysaccharide (LPS), which consists of three main components: lipid A, the membrane-anchored portion responsible for the molecule’s endotoxic effects; the core oligosaccharide, comprising both inner and outer regions rich in sugars; and the O-antigen (or O-polysaccharide chain), the highly variable, surface-exposed region that contributes to the antigenic specificity of the bacterium [39,40]. The O-antigen is essential for the appropriate production or function of other outer membrane virulence factors, which may account for the diminished efficiency of Ysc-mediated host cell internalization in its absence [39,40]. *Y. enterocolitica* has over 70 serotypes defined by O antigens, and when combined with its six biotypes, these form distinct bioserotypes, some of which (e.g., 1B/O:8, 4/O:3) are strongly linked to pathogenicity [41,42]. Flagella and thus motility play a crucial role in commencing host cell invasion before *Y. enterocolitica* enters into close contact with the intestinal epithelium. The flagellar regulatory genes are *flhDC* (the master regulatory component) or *fliA* [43]. Yersinia must attach to the host cell surface and remain close during the delivery phase to efficiently transport the YadA protein. This protein was previously called YopA (Yersinia outer membrane protein), which mediates mucus and epithelial cell adhesion and enhances host cell invasion [22,44]. YadA is a multifunctional, surface-exposed virulence factor that mediates adhesion to extracellular matrix proteins and is encoded by the structural *yadA* gene located extrachromosomally on the pYV plasmid [45]. YadA expression is strongly induced at or above 37 °C, and under these conditions, it becomes so abundant that it can nearly coat the entire outer surface of the bacterial cell. Despite its high effectiveness and abundance, YadA is surprisingly not widely utilized. This limited usage is due to a single-nucleotide deletion that causes a frameshift mutation, disrupting its expression in many strains [22,44]. YadA also elicits an inflammatory response in epithelial cells by inducing the production of interleukin-8 (IL-8), a process mediated by mitogen-activated protein kinases (MAPKs). Among the various virulence factors of *Y. enterocolitica*, YadA appears to play the most critical role, contributing to adhesion, invasion, and resistance to serum-mediated killing [22,44].

**Figure 1:**
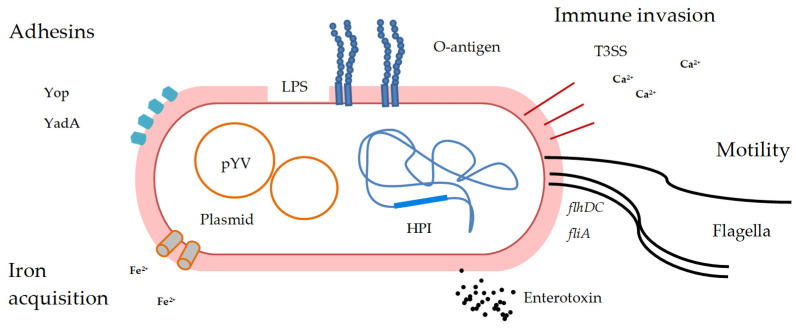
*Yersinia enterocolitica* with representation of virulence factors like the Yersinia adhesion A protein (YadA), virulence plasmid (pYV), and T3SS (Type III Secretion System) requiring Ca^2+^ for activation, LPS (lipopolysaccharide), O-antigen: part of LPS; important for immune evasion. Iron acquisition, essential for bacterial survival in host environments. Flagella enable motility controlled by *flhDC* and *fliA* regulatory genes. HPI (High-Pathogenicity Island) encodes siderophores and iron uptake systems (yersiniabactin), contributing to pathogenicity.

All human–pathogenic Yersinia species harbor a conserved 70-kb virulence plasmid (pYV) that plays a pivotal role in evading the host’s innate immune system. Strains belonging to biovar 1A are considered nonpathogenic because biovar 1A strains lack the pYV plasmid and the major chromosome determinants of virulence. The biovar 1A strains of *Y. enterocolitica* are distributed globally and have been isolated from asymptomatic and symptomatic individuals [46]. This pYV plasmid enables the bacteria to replicate and disseminate extracellularly, avoiding phagocytosis and inflammatory responses. A key set of genes encoded on this plasmid becomes transcriptionally activated at 37 °C in the presence of millimolar concentrations of calcium, conditions that mimic the environment within a mammalian host. These genes encode components of the Type III Secretion System (T3SS), a sophisticated needle-like “nanomachine” known as the injectisome. This structure penetrates host cell membranes, allowing the delivery of effector proteins directly into the host cytoplasm. The translocon, another essential component, forms a pore in the host membrane to facilitate the passage of these effector proteins. Once inside the host cell, these effectors disrupt immune signaling pathways, inhibit phagocytosis, and promote bacterial survival and proliferation [47]. *Y. enterocolitica* also produces Yersinia stable toxin (Yst), a thermostable, chromosomally encoded enterotoxin implicated in the induction of diarrheal symptoms in humans. This toxin contributes to fluid secretion in the intestinal tract, playing a key role in the pathogenesis of yersiniosis. Interestingly, non-invasive *Y. enterocolitica* strains belonging to biotype 1A, which are typically considered less virulent, frequently harbor a variant of the *yst* gene. In these strains, the presence of *yst* may represent the primary, or even sole, virulence determinant responsible for the diarrheal illness they cause [43]. A less-known type II secretion system (T2SS) probably plays a dual role for both the pathogenicity and the environmental survival of *Y. enterocolitica* [48]. Similar to the Ysa Pathogenicity Island (Ysa-PI), the High-Pathogenicity Island (HPI) is found exclusively in highly virulent *Y. enterocolitica* strains belonging to biotype 1B. The HPI is classified as an iron-capture island because most of its genes are involved in the biosynthesis, transport, and regulation of the siderophore yersiniabactin. This iron-chelating molecule enables the bacterium to scavenge iron from the host environment, an essential function for survival and virulence under the iron-limited conditions typically encountered during infection [23]. Figure 1 presents the main virulence factors of pathogenic *Y. enterocolitica*.

### Role of Key Virulence Genes

*Y. enterocolitica* possesses various virulence factors that enable it to evade host defenses and cause infection. *Y. enterocolitica* possesses several key virulence genes that contribute to its pathogenicity, such as *ail*, *inv*, and *ystA* [8,26,49]. The *ail* gene is always present in pathogenic serotypes and plays a role in serum resistance and invasion of host cell borders [25]. The *inv* gene, present in both pathogenic and non-pathogenic strains, is involved in cell invasion [8]. The *ystA* gene is common in pathogenic biotypes and can be used to identify them, while *ystB* is found in non-pathogenic biotypes [26,50]. In a molecular epidemiology study, in contrast to pathogenic isolates of other biotypes, yersiniabactin was present in biotype 1B isolates [51]. Biotype 1A strains, although traditionally considered non-pathogenic, show evidence suggesting that *ystB* may play a role in the pathogenicity of this biotype [46]. Other virulence genes include *yadA* and *virF*, which are plasmid-borne and present in most pathogenic strains such 5/O:3 and 2/O:9 [26]. Other genes such as *sat* (streptogramin acetyltransferase), *fepD* (enterochelin transporter, ABC), *fes* (enterochelin esterase), and *ymoA* (Yersinia modulating protein) can be found in both pathogenic biotypes and biotype 1A strains of *Y. enterocolitica* [26]. However, not all pathogenic *Y. enterocolitica* strains carry all traditional virulence genes, suggesting the presence of unknown virulence markers [26]. A recent study contributes to understanding the possible role of *Y. enterocolitica* biotype 1A as a human pathogen [7]. Consistent with this, the biotype 1A strains identified from human, pig and wild boar isolates lacked the major virulence genes *ail*, *yadA*, and *virF*; however, all 14 strains identified as *Y. enterocolitica* 1A by MALDI–TOF carried the *ystB* gene [52].

## 4. Prevalence of *Y. enterocolitica* in Domestic and Wild Pigs

Pure Eurasian wild boars and their hybrids with domestic pigs can be found in the wild on most continents. These wild pigs are known to carry a significant number of zoonotic and epizootic pathogens, including *Y. enterocolitica* [53].

*Y. enterocolitica* is prevalent in both domestic pigs and wild boars, with domestic pigs considered the primary reservoir for human infection [54]. In several studies, distinct strains were identified in wild boars compared to domestic pigs, with bioserotypes 4/O:3, 2/O:9, and 2/O:5,27 detected in wild boars, whereas bioserotype 4/O:3 was predominant in domestic pigs [55,56,57]. The first pathogenic *Yersinia enterocolitica* 4/O:3 strain has been identified in hunted wild boars in Poland, raising potential public health concerns [58]. However, a German study found mostly biotype 1A *Y. enterocolitica* in wild boars, with no enteropathogenic bioserotypes 4/O:3 or 2/O:9 identified [59].

In the Czech Republic study, seroprevalence of *Y. enterocolitica* reached 78.7% in domestic pigs and 65.9% in wild boars [60]. In the United States, a large-scale survey across 15 states found pathogenic *Y. enterocolitica* in 13.1% of pig fecal samples [61]. Prevalence rates vary between studies and sample types, with tonsils yielding higher prevalence (38.4%) compared to feces and carcass surfaces [54]. Prevalence also varies across production phases, with a prevalence of 5.1% in United States herds, with higher rates in finishing pigs (10.7%) and gestating sows (9.1%) [62]. A Dutch study reported lower prevalence rates of 9.3% in porcine tonsils and 3.3% in pig feces [57]. Studies in Poland revealed high contamination rates in roe deer (60%), red deer (43.8%), and wild boar (55%) carcasses [63]. Another Polish study found *Y. enterocolitica* in 25.4% of examined game animals, with wild boars being the most common carriers [13]. In Sweden, the prevalence of wild boars was 31% [53], and in Spain, seroprevalence reached 52.5%, with tonsil detection at 33.3% with real-time PCR. The study found that *Y. enterocolitica* and *Y. pseudotuberculosis* were prevalent among wild boars in the Basque Country, with *Y. enterocolitica* being the most common. The risk of infection among wild boars was influenced by the season and the area in which they live [64]. In Italy, a study found that *Y. bercovieri* was more prevalent than *Y. enterocolitica* in wild boars, with a prevalence rate of 35.4% [65]. The presence of virulence genes in wild boar isolates (e.g., *ystB*, *ymoA*, *ail*) and the increasing antimicrobial resistance over time raise public health concerns [66]. In wild boars hunted in 2016 in Finland, seroprevalence was significantly higher in adult than in young animals, indicating infection or reinfection at an older age [67].

Wild boars’ growing population and their proximity to urban and agricultural areas increase the risk of zoonotic transmission through game meat and environmental contamination [66]. These findings highlight the potential risk of *Y. enterocolitica* transmission from game meat and pork products to humans. Environmental contamination correlates with fecal prevalence, but the environment is not the primary source of infection [68].

### Geographic and Seasonal Trends of Y. enterocolitica

*Y. enterocolitica* presents a worldwide distribution in pigs. Geographic distribution in China indicated a negative correlation between *Y. enterocolitica* prevalence in pigs and factors such as elevation and annual average air temperature but a positive correlation with annual precipitation [16,69]. In wild boars in the Basque Country, Spain, the highest antibody levels and *Y. enterocolitica* prevalence were observed in mountainous areas at altitudes higher than 600 m, with very cold winters, and the highest annual rainfall for each dominant climate. Moreover, areas with high ovine populations had the highest prevalence of *Y. enterocolitica* [64]. A similar trend was observed in pigs slaughtered in China, in which the incidence of *Y. enterocolitica* was higher in colder areas with higher annual precipitation than in warm areas [16,69]. Interestingly, contrary to the common belief that *Y. enterocolitica* is more prevalent in winter, a French study found significantly higher prevalence during the warm period, with 13.7% of pigs testing positive [70]. In Finland, a higher prevalence was observed in intestinal samples during July and August, while tonsil samples showed no seasonal variation [71]. Seasonal variation in the prevalence of *Yersinia* species in milk and milk products in Chennai, India, was observed, with the highest incidence (40%) during the cold season and the lowest (5%) during the hot season [72].

## 5. One Health Relevance

The One Health approach is necessary to elucidate the routes of transmission of *Y. enterocolitica* and consequently inform targeted interventions for the prevention and management of yersiniosis [38]. Among many foodborne agents, *Y. enterocolitica* is an emerging, versatile foodborne zoonotic pathogen that can result in high morbidity and mortality, especially in infants and young children [73,74]. Acute yersiniosis commonly presents in children and adolescents as acute, self-limiting bloody diarrhea that can last 1 to 3 weeks. Acute yersiniosis commonly presents in children and adolescents as acute, self-limiting bloody diarrhea that can last 1 to 3 weeks. Children younger than 5 years are at higher risk of contracting the disease and developing complications such as mesenteric lymphadenitis and extraintestinal infections [75,76]. Necrotizing enterocolitis has also been described in infants [75]. Human infection typically manifests as acute diarrhea, mesenteric adenitis, terminal ileitis, and pseudoappendicitis, and in rare cases, it may even lead to sepsis [76]. Reactive arthritis has been reported as a complication following *Y. enterocolitica* enteritis [77]. Bacteremia is prominent in the setting of immune suppression or in patients with iron overload or those being treated with desferrioxamine [18]. The risk of invasive disease increases under conditions that predispose exposure to iron overload, such as in cases of thalassemia, hemochromatosis, and transfusion-associated infections [75].

Over the past decade, the incidence of yersiniosis, particularly cases requiring hospitalization, has increased significantly. According to the European Union (EU) One Health 2023 Zoonoses report, yersiniosis was the fourth most frequently reported foodborne zoonosis in the EU in 2023, following campylobacteriosis, salmonellosis, and Shiga toxin-producing *Escherichia coli* (STEC) infections. In 2023, EU countries reported 8738 confirmed cases of human yersiniosis, resulting in a notification rate of 2.4 cases per 100,000 population. Yersinia infections increased (*p* < 0.01) during the 2019–2023 period, with meat and meat products being the only category testing positive in 2023, at a rate of 10.2%. Currently, *Y. enterocolitica* exhibits a higher prevalence and is recognized as the most significant genus of *Yersinia* in swine populations [4]. In recent years, increasing numbers of *Y. enterocolitica* outbreaks have also been linked to vegetables in addition to pork products. Good agricultural and hygiene practices in food storage and processing, as well as proper washing and peeling of vegetables in home kitchens, can decrease the risk of contamination of fresh produce and prevent further infections [78,79].

### Foodborne Outbreaks (FBO)

*Y. enterocolitica* is recognized as a significant biological hazard in the EU, and in comparison, *Y. pseudotuberculosis* appears to have limited importance as a reservoir species in pigs [4]. The latest data from EFSA reported at least 16 FBOs caused by *Y. enterocolitica* in EU Member States, along with one additional outbreak reported by a non-Member State, Switzerland [4]. Yersiniosis has been a notifiable disease in New Zealand since 1996, and the country reports a relatively high rate of yersiniosis compared to other developed nations. Over 99% of human cases of Yersiniosis in New Zealand are nowadays attributed to *Y. enterocolitica;* however, the precise sources and transmission routes of infection have remained unclear for a long time [38].

Table 1 presents human infections of *Y. enterocolitica* due to sporadic cases and the few reported outbreaks.

Human infections due to *Y. enterocolitica* mainly occur as sporadic cases. However, a few outbreaks have been reported worldwide of various bioserotypes, such as 4/O:3 in Sweden and Denmark [95], 2/O:9 in Norway [91], B1/O:8 in the United States [41], and 3/O:3 in China [90]. Currently, the most common bioserotypes of *Y. enterocolitica* isolated from human clinical samples within the EU (European Food Safety Authority (EFSA) and European Centre for Disease Prevention and Control (ECDC) were 4/O:3 (86.9%) and 2/O:9 (10.7%) [4]. In the United States, serogroup O:8 continues to be the most prevalent [76].

In the spring of 2019, the Swedish Public Health Agency and Statens Serum Institut in Denmark independently identified an FBO caused by *Y. enterocolitica* 4/O:3 that, after sequence comparison, turned out to be a cross-border outbreak [23]. The traceback investigation suggested shipments of fresh prewashed spinach from Italy as a common source for the cross-border outbreak [42,95]. In Australia, an FBO in a residential aged care facility was linked to nutritional milkshakes, highlighting that biotype 1A can be pathogenic, particularly in vulnerable populations, although with generally mild symptoms [10]. In 2023, no RTE samples were taken from previously tested categories such as salads, processed foods, and prepared dishes. Despite the known consumer risk due to the absence of cooking steps before consumption, only a few Member States submitted data on RTE foods [78,96]. Regarding non-RTE foods, six Member States submitted 1210 samples in 2023, with 99.4% from meat and meat products. Among these, 10.2% tested positive for Yersinia, with the highest rate observed in fresh pork (12.6%). Milk and milk products were not sampled in 2023, although they had shown the highest contamination rate (11.0%) during 2019– 2022, followed by meat and meat products (6.2%) [4]. A total of 17 FBOs caused by Yersinia were reported in 2023, representing an increase of three outbreaks compared to 2022. These outbreaks occurred in seven Member States (Austria, France, Germany, Poland, Slovakia, Spain, and Sweden). *Y. enterocolitica* was identified as the causative agent in 16 of the outbreaks, while the Yersinia species was not specified in one case. Additionally, one outbreak caused by Yersinia was reported by a non-Member State (Switzerland) [4].

## 6. Farm-to-Fork Transmission

### 6.1. Routes of Transmission of Yersinia enterocolitica in Pigs

The transmission of *Y. enterocolitica* in pigs occurs primarily through animal-to-animal contact and environmental sources [97,98]. Pigs are naturally burrowing animals, and *Yersinia* infection typically occurs through inhalation or ingestion of the bacteria via the snout or mouth. Domestic pigs are usually infected during the fattening period, and their seroprevalence decreases with age, being low in sows [67]. Experimental infection models in Large White pigs have shown that *Y. enterocolitica* can cause persistent colonization regardless of whether the inoculation is oral or nasal, suggesting that infection may occur through both the mouth and the nasal cavities [99]. After the pathogen colonizes pigs through oral or nasal routes, it initially appears in tonsils and feces before expanding to the digestive system and extraintestinal organs [100]. The bacterium colonizes the pig intestine, causing minimal inflammation due to reduced IL-8 and increased IL-10 production in porcine macrophages [101]. *Y. enterocolitica* O:3 demonstrates unique virulence properties, including improved long-term colonization in pig intestines and modulation of the porcine immune response [101]. The introduction of infected pigs into herds significantly contributes to the spread of *Y. enterocolitica* between farms [102]. The prevalence of pathogenic *Y. enterocolitica* in pig herds varies widely, from 0% to 100%, influenced by farm-specific factors [54,103]. Farm-specific factors, such as drinking from nipples [104], absence of coarse feed or bedding, and restricted pest animal access, are associated with higher *Y. enterocolitica* prevalence [105]. Risk factors for *Y. enterocolitica* infection in pigs include fattening farms, longer lairage periods, and the winter season [100]. Vanantwerpen et al. [106] studied the link between microbiological and serological diagnosis of *Yersinia* spp. in pig batches and the use of serology to classify batches and reduce carcass contamination risk.

A United States study on swine, with an overall on-farm prevalence of 45.1% (55/122 farms), found higher prevalence associated with vaccination against *Escherichia coli*, increased mortality from scours, and inclusion of meat or bone meal in the grower–finisher diet [107]. Occupational contact with pigs and consumption of raw or undercooked pork are significant risk factors for human infection [108]. Other studies have found that *Y. enterocolitica* is more frequently detected in fresh fecal samples than in manure pits [109], reflecting a decline in bacterial viability or detectability over time. Protective factors for pigs include using municipal water, organic production, and certain feed manufacturers [102]. Farm-level factors associated with higher prevalence include multiple piglet suppliers, high density of pig farms in the area, and semi-slatted floors in fattening units [110]. Implementing proper biosecurity measures, reducing piglet suppliers, and prohibiting pets in stables could help lower *Y. enterocolitica* prevalence in pigs at slaughter [110].

The invasin protein (InvA) plays a crucial role in colonization and persistence of *Y. enterocolitica* in pigs, with higher InvA expression improving colonization [111]. While a plasmid is key to *Y. enterocolitica* pathogenicity, intestinal wall penetration may be governed by chromosomal genes [112]. Pathogenic strains typically carry the chromosomal *ail* gene, which plays a key role in bacterial adhesion to and invasion of host cells, as well as in conferring resistance to serum-mediated killing. However, the presence of the *ail* gene alone is not sufficient to reliably predict the pathogenic potential of Yersinia isolates [113]. *Y. enterocolitica* pathogenicity is largely attributed to a plasmid, although chromosomal genes may also play a role in intestinal wall penetration [112]. While enterotoxin production does not directly contribute to pathogenesis, it may promote bacterial proliferation and shedding [112]. The epidemiology of *Y. enterocolitica* infections remains complex, with most cases occurring sporadically [6]. Within-batch prevalence in positive farms can range from 5.1% to 64.4% [110]. These findings highlight the widespread presence of pathogenic Yersinia in slaughter pigs and the potential risks for public health. A seroepidemiological study conducted in Chiba Prefecture, Japan, found that 37.8% of domestic pigs from seven regions were seropositive for pathogenic *Yersinia*. The pigs were tested using an ELISA based on plasmid-encoded *Yersinia* outer membrane proteins (Yops) antigens. These findings indicate that pathogenic *Yersinia* is widely distributed among the pig population in the region, underscoring the potential role of pigs as a source of human yersiniosis in Chiba [114]. Epidemiological studies suggest that limiting contact between infected and non-infected herds can reduce herd prevalence of zoonotic *Y. enterocolitica* [101].

#### 6.1.1. Slaughter of Pigs

Pigs are considered the main reservoir for human pathogenic *Y. enterocolitica*, with contamination of carcasses and pluck sets originating from infected pigs on farms [105]. In Croatia, the prevalence of *Y. enterocolitica* in pig tonsils varies across different farm types, ranging from 29% to 52% [55]. Contamination can occur during slaughter, leading to carcass contamination and introduction into the food chain [8]. Critical control points during slaughter include intestine removal, tonsil excision, and head deboning [115]. While carcass contamination decreases after chilling, *Y. enterocolitica* can persist in the slaughterhouse environment [54,115]. Studies in Germany and Finland reported tonsil prevalence rates of 60% [71,116]. The pathogen is also found in other offal, including tongues, lungs, and livers, with tonsils likely serving as a source of contamination during slaughter [116]. Direct plating methods have proven effective for detection and enumeration, with average concentrations of 4.5 log_10_ CFU/g in tonsils [117]. *Y. enterocolitica* is prevalent in swine populations, with studies showing 92.2% of slaughter batches containing at least one infected pig [118]. The prevalence increases as pigs mature, with finishing pigs having the highest rates at 10.7% compared to suckling piglets (<1%) and nursery pigs (1.4%) [62]. *Y. enterocolitica* was detected more frequently in the colon content samples of both finishing pigs (11.9%) and piglets (8.6%) compared to other sample types [119]. The pathogen was not isolated from piglets and weaners in fecal samples but was found during the fattening stage, with prevalence ranging from 0 to 65.4% [54]. Pathogenic strains, defined by the presence of the *ail* gene, were found in 28.2% of slaughter lots [7] and 3.80% of fecal samples across multiple states [118]. Serotypes O:3 and O:5 are both predominant in pigs, with O:3 being more common [120]. In a study conducted in Sardinia, an island in the Mediterranean Sea, most isolates belonged to bioserotype 4/O:3, with the *ail* virulence gene detected in all of them [119]. In Finland, contaminated pig offal has been identified as a major vehicle for *Y. enterocolitica* transmission from slaughterhouses to humans [116]. However, improved slaughtering methods, including enclosing the anus in a plastic bag after rectum-loosening, can reduce yersiniosis prevalence. In Norway, most fattening pigs are slaughtered between 150 and 180 days of age. By this stage, the tonsils may present a greater risk for harboring human–pathogenic *Y. enterocolitica* than the intestinal contents, as the prevalence in the latter tends to decline by the time of slaughter. Consequently, strict hygienic handling of the head and plucks is essential to prevent carcass contamination. One of the most effective control measures may be early decapitation, with the head, tongue, and tonsils removed on a separate processing line [101]. Avoiding incision of the submaxillary lymph nodes may also help reduce spread. However, under European Regulation (EU) 2019/627 on official controls, in force since 14 December 2019, the incision and examination of the submandibular lymph nodes (*Lnn. mandibulares*) is no longer mandatory [121]. This targeted approach helps reduce the risk of cross-contamination and disease spread.

#### 6.1.2. Meat Consumption

*Y. enterocolitica*, particularly bioserotype 4/O:3, is a zoonotic pathogen that causes yersiniosis in humans, with pigs serving as the primary reservoir [122,123]. In 2023, Yersinia was detected by six Member States in more than eight different animal categories, which overall include more than 30 animal species. The majority of the units tested in the EU (n = 6901) were from cattle, and the proportion of positives was 0.97% for *Y. enterocolitica* and 1.0% for *Y. pseudotuberculosis*. The proportions of positive sampling units from ‘small ruminants’, pigs, and ‘pet animals’ were 0.60%, 1.5%, and 1.4% for *Y. enterocolitica* and 0.25%, 0% and 2.9% for *Y. pseudotuberculosis*, respectively [4]. The proportion of positive samples for *Y. enterocolitica* was highest in pigs (1.5%), followed by cattle (0.97%) and ‘small ruminants’ (0.60%) tested in animals in 2023 [4]. Many cases of human *Y. enterocolitica* infections are related to the consumption of undercooked contaminated pork or cross-contamination of other food items during the handling and preparation of raw pork [4].

Pork should only be consumed after thorough cooking, especially when given to young children. Proper kitchen hygiene is required to avoid cross-contamination. Prolonged cold storage of contaminated food allows the survival and growth of Yersinia. However, contaminated vegetables have also been identified as sources of infection, highlighting the importance of proper food handling and cooking practices [4,79]. The ability of Yersinia bacteria to survive and grow at low temperatures underscores the need for thorough cooking of food, especially pork, and proper hygiene practices in food preparation to prevent yersiniosis [4]. Pork has been identified as the commodity with the highest levels of contamination and is the only source of *Y. enterocolitica* biotype 4 isolates [7]. Additionally, these biotype 4 isolates are clonally related to human clinical isolates, which confirms that raw pork poses a risk for exposure and infection with pathogenic *Y. enterocolitica* [124].

Previous studies employing whole-genome sequencing (WGS) methodologies have identified closely related clusters of *Y. enterocolitica* isolated from raw pork and from diseased humans. This strongly suggests that transmission to humans occurs through pigs and contaminated food [125,126]. Moreover, three *Y. enterocolitica* biotype 1A isolates each matched a human clinical isolate from a previous study by Stevens et al. [7], showing that pork and poultry meat represent a risk of infection for humans.

#### 6.1.3. Swine Meat Products

A study in four South African cities analyzed 581 retail meat samples of pork and beef—292 raw intact, 167 raw processed, and 122 RTE foods. Contamination with *Y. enterocolitica* was found in 15% of raw intact, 11% of raw processed, and 7% of RTE products. These findings indicate that retail beef, pork, and meat products in South Africa may carry potentially pathogenic *Y. enterocolitica*. In most cases, bioserotype 1A/O:8 was identified, and these strains were found to harbor various virulence genes associated with human yersiniosis, indicating a potential public health risk [127]. The prevalence of *Y. enterocolitica* was particularly high in tripe (27%, n = 7), followed by bone or skeleton tissues (18%, n = 6), organs (16%, n = 9), muscles (12%, n = 21), and processed meat samples (10%, n = 25). In contrast, biltong exhibited a significantly lower contamination level (5%, n = 2). No statistical difference (*p* = 0.0758) was reported among the different sample types [127]. *Y. enterocolitica*, particularly bioserotype 4/O:3, is prevalent in pigs and can contaminate carcasses during slaughter [54,105,118]. The prevalence of pathogenic *Y. enterocolitica* in pig herds varies widely, from 0% to 100%, influenced by farm-specific factors [54,105]. The pathogen is also common in pork products, with a study in Finland reporting 92% prevalence in pig tongues and 25% in minced meat using PCR detection [128]. Most isolates belong to biotype 1A, but potentially pathogenic strains carrying virulence genes like *ail* and *yadA* have been identified [13]. A study from Malaysia identified *Y. enterocolitica* strains belonging to three bioserotypes, namely 3/O:3, 1B/O:8, and 1A/O:5, isolated from pigs and pig-derived products. Notably, 90% of the strains were multidrug-resistant. Among them, the 3/O:3 strains exhibited greater genetic heterogeneity compared to the other bioserotypes. Moreover, all 3/O:3 isolates carried the pYV virulence plasmid and tested positive for 11 out of the 15 virulence genes assessed (*hreP*, *virF*, *rfbC*, *myfA*, *sat*, *inv*, *ail*, *ymoA*, *ystA*, *tccC*, and *yadA*), highlighting their pathogenic potential [129]. Meat and fresh produce samples collected at the retail level in Switzerland for the presence of *Y. enterocolitica* were subjected to WGS on the recovered isolates. Pork exhibited the highest contamination rate and was the only commodity existing in conjunction with *Y. enterocolitica* bioserotype 4 [7]. Notably, the biotype 4 isolates were clonally related to strains obtained from human clinical cases, reinforcing the role of raw pork as a significant source of exposure and infection with pathogenic *Y. enterocolitica* [4,124,125]. Previous studies employing WGS have similarly identified closely related clusters of *Y. enterocolitica* from raw pork and human clinical isolates, supporting the hypothesis of transmission from pigs to humans through foodborne routes [38,51].

### 6.2. Zoonotic Potential and Foodborne Transmission to Humans

The first shotgun genome sequence of a microbial pathogen from the Philippines was *Y. enterocolitica* subsp. *palearctica* strain PhRBD_Ye1, isolated from swine, underscoring the role of pigs as a natural reservoir for yersiniosis and revealing close genetic relatedness to a human clinical isolate from Germany [130]. Complete genome sequences of *Y. enterocolitica* human isolates include strain 8081, a European pathogenic type 1B strain (1B/O:8) [131]; strain 105.5R(r), a type 3 strain (3/O:9) from China [132]; and strain Y11, a type 4 strain from Germany (4/O:3) [133,134]. A shotgun sequence has been generated for a fourth strain, W22703, a biotype 2 strain (2/O:9) from Germany [135]. Analysis of PhRBD_Ye1 demonstrates that it is of type 4 and most closely related to strain Y11, the human isolate from Germany, of subspecies palearctica [133]. Despite lacking several virulence factors found in the type 1B strain 8081, PhRBD_Ye1 carries the *ail* gene, a key marker of pathogenicity in *Y. enterocolitica*. It also contains the gene cluster for O-antigen synthesis typical of O:3 isolates, suggesting it is likely a type 4/O:3 strain [130].

Several recent studies have established the close clonal relatedness of human clinical isolates to *Y. enterocolitica* biotype 4 isolated from pork in Brazil [136], to *Y. enterocolitica* biotype 2/3 from pork and biotype 1A from seafood in New Zealand [125], and to *Y. enterocolitica* biotype 1A from seafood collected in Germany [137]. Currently, biotype 4 is the most common *Y. enterocolitica* isolated from human clinical samples within the EU (European Food Safety Authority (EFSA) and European Centre for Disease Prevention and Control (ECDC), 2024) [4]. Risk factors for human infection include occupational contact with pigs and consumption of raw or undercooked pork [107]. *Y. enterocolitica* is mainly transmitted through contaminated food and water, with pigs being a significant reservoir [36]. Multidrug-resistant strains of *Y. enterocolitica* 4/O:3 have been identified, particularly in pigs from large integrated farms [55]. The drugs of choice are aminoglycosides or trimethoprim–sulfamethoxazole [77]. Severe cases may require treatment with fluoroquinolones or third-generation cephalosporins [23]. High antimicrobial resistance frequency was observed for ampicillin (94%), cephalothin (83%), and amoxicillin (41%) from meat in South Africa [127], and multidrug-resistant isolates from pigs and human samples were also observed [136].

## 7. Prevention and Control of *Y. enterocolitica*

Control measures at both the farm and slaughterhouse levels play a pivotal role in mitigating the risks associated with *Y. enterocolitica* in the pork production chain [20]. However, controlling *Y. enterocolitica* requires a multifaceted approach addressing various points in the food production and consumption chain.

### 7.1. Prevention and Biosecurity on Pig Farms

Existing pig farming systems differ significantly in terms of biosecurity levels and could, therefore, pose differing animal health risks [55]. On farms, effective biosecurity protocols including controlled access to pig housing, proper sanitation of equipment, and restrictions on animal movement are fundamental in preventing the introduction and spread of pathogens. Additionally, routine hygiene practices and targeted surveillance programs are critical for early detection and management of infections [20,55]. The occurrence of *Y. enterocolitica* in swine varies significantly between farms, suggesting the presence of underlying factors that influence its prevalence in farm environments [20,138]. Among these, the management system is considered a key determinant in controlling the transmission of pathogenic *Y. enterocolitica* within pig herds. Colostrum is considered a protective factor. Piglets born by Cesarean section without colostrum showed higher colonization by *Y. enterocolitica* compared to naturally born piglets that received it. However, seropositivity alone is not a reliable indicator of prevalence, as antibodies detected at birth likely result from maternal transfer via colostrum, and these levels decline with age [139]. Other important aspects of biosecurity on pig farms include preventing the transmission of *Y. enterocolitica* at the interface between livestock and wildlife, as well as understanding the role that wild and peridomestic rodents play as sources of this zoonotic pathogen for pigs [140]. The common practice of mixing pigs from different groups is widely recognized as a major risk factor for the spread of this pathogen [141]. In farrow-to-finish farms, where piglets are not sourced from external suppliers, the risk of introducing infections is inherently lower. In contrast, in fattening farms, the number of piglet suppliers has been identified as a significant risk factor for the introduction and spread of pathogenic *Yersinia* spp. [102]. The likelihood of purchasing infected piglets, and consequently spreading the pathogen within pens, increases as the number of different suppliers rises [142]. Furthermore, when pigs are transferred to facilities that do not follow the all-in/all-out system, the infection can rapidly disseminate throughout the entire population [140]. Other risk factors for the dissemination of the pathogen include the presence of semi-slatted floors in fattening pig units [110]. These flooring systems allow organic matter to accumulate in the solid areas, creating reservoirs for pathogenic microorganisms such as *Y. enterocolitica*. The type of housing system can play a significant role in prevalence confirmed as a study conducted in Germany with conventional pig farming systems showed a higher proportion of *Y. enterocolitica*-positive pigs (29%) compared to organic systems (18%), with twice as many positive tonsil samples (22% vs. 11%) [143]. This difference may be attributed to several factors: conventional systems typically involve higher stocking densities, less outdoor access, and greater stress levels, all of which can compromise immune function and facilitate horizontal transmission of pathogens.

Despite these efforts, significant challenges remain in identifying and controlling *Y. enterocolitica*, particularly due to its ability to colonize healthy, asymptomatic pigs, which can act as reservoirs and contribute to silent transmission within herds [103]. Current intervention methods at the farm level are often insufficient to eliminate the pathogen, highlighting the need for more effective strategies and further research into animal-level interventions and potential vaccination programs [103]. At the slaughterhouse level, contamination can occur during processing, particularly during evisceration and carcass handling. While improved hygienic practices, such as equipment sterilization, controlled workflow design, and staff training, help minimize cross-contamination, they are not entirely effective in eradicating *Y. enterocolitica* from the processing environment [103]. In this context, standardized surveillance strategies are critical. Regular microbiological testing and risk-based monitoring protocols can enhance the early detection of *Y. enterocolitica* and inform timely responses. Comprehensive and harmonized control measures, encompassing the entire pork production continuum, from farm biosecurity to slaughterhouse sanitation, are essential to reduce public health risks and ensure food safety [9]. Coordinated efforts involving farmers, veterinarians, food safety authorities, and public health agencies are necessary to effectively manage this zoonotic threat.

### 7.2. Prevention of Y. enterocolitica Infection in Humans

Human infection rates with *Y. enterocolitica* have varied over time, with the highest incidence consistently reported in children under 5 years of age, who are particularly susceptible due to immature immune responses and increased exposure in communal environments such as childcare centers [144]. Seasonal peaks during winter are linked to the psychrotrophic nature of the bacterium, which allows survival and multiplication at refrigeration temperatures, thereby extending its persistence in the food chain [144]. Prevention strategies target the pork production continuum, as pigs serve as the principal reservoir for pathogenic bioserotypes (notably 4/O:3 and 2/O:9), with control measures encompassing improved on-farm biosecurity, enhanced slaughter hygiene, and interventions to prevent carcass contamination, although cost-effective and widely adopted solutions remain limited [144]. In childcare settings, outbreaks are mitigated through strict hygiene practices, environmental disinfection, prompt isolation of symptomatic individuals, and food safety controls, while vaccine development—though promising in animal models—has not yet yielded an approved human formulation, warranting further translational research [144]. Host genetic predisposition, particularly carriage of the HLA-B27 allele, is strongly associated with the development of reactive arthritis and other post-infectious sequelae following *Y. enterocolitica* infection; elucidating such genetic risk factors could enable targeted prevention and clinical monitoring of vulnerable populations [145]. Accurate surveillance depends on sensitive detection methods, as traditional culture-based techniques may underestimate prevalence due to slow growth and low bacterial loads in complex matrices. Standardized DNA-based approaches, such as quantitative PCR assays targeting virulence-associated genes (*ail*, *ystA*, *inv*), offer greater sensitivity and specificity for both food and environmental samples, thereby improving risk assessment and outbreak investigations [145]. At the consumer level, education on safe handling and preparation of pork products remains critical, as does prevention of cross-contamination in the kitchen and maintenance of appropriate refrigeration to inhibit bacterial growth.

## 8. Knowledge Gaps and Future Directions

This review highlights the critical need for harmonized and standardized surveillance protocols to accurately estimate the prevalence of *Y. enterocolitica* in swine populations, thereby providing a robust foundation for evidence-based control and prevention strategies. Recent research highlights the need for integrative One Health approaches to address *Y. enterocolitica* infections. New Zealand has seen an emergence of *Y. enterocolitica* biotype 2/3 serotype O:9, emphasizing the importance of whole-genome sequencing in epidemiological investigations [38,125]. Phage therapy shows promise, with the Yersinia phage X1 demonstrating efficacy in reducing *Y. enterocolitica* infection in mice and lowering proinflammatory cytokine levels [146]. Adopting a global One Health framework, integrating human, animal, and environmental data, together with WGS, will be critical for mapping major transmission pathways. WGS enhances outbreak detection, strengthens surveillance, and enables in-depth genomic comparisons between international isolates, supporting the identification of endemic clones and the tracking of emerging strains. Effective surveillance and control measures are crucial to reducing the risk of *Y. enterocolitica* infection in both animals and humans. The meat sector could benefit from a control plan that classifies herds through serological testing and integrates these results into risk-reduction strategies, though cost–benefit evaluations remain necessary. The seemingly low prevalence detected in food may reflect the limitations of current selective culture methods, which often lack sensitivity. This highlights the need for standardized, sensitive, and rapid methods for detecting *Y. enterocolitica* in clinical, food, and environmental samples.

## Figures and Tables

**Table 1:** Foodborne outbreaks (FBOs) of infection with *Yersinia enterocolitica*.

Year	Location	Month	Cases (No.)	Sevorar	Source	Reference
1976	New York	September	38	O:3	Flavored milk	Black et al. [80]
1980	Japan	April	1051	O:3	Pork	Maruyama et al. [81]
1988–1993	New Zealand	-	918	4/O:3	-	Fenwick et al. [82]
1989	Georgia	November	15	O:3	Pork chitterlings (intestines)	Lee et al. [83]
1995	Vermont and New Hampshire	October	10 (1 fatal)	O:8	Bottles of pasteurized milk from a local dairy	Ackers et al. [84]
2004	Japan	July	42	O:8	Salads with ham	Sakai et al. [85]
2005–2006	Norway	December–February	11	2/O:9	Processed pork	Grahek-Ogden et al. [86]
2006	Japan	July	3 (family)	2/O:9	Contaminated food, such as pork (speculated)	Moriki et al. [87]
2009–2010	Germany	-	563	O:3 (93.6%), O:9 (5.1%) O:5,27 (0.4%)	Raw minced pork	Rosner et al. [88]
2010–2015	China	-	7304	3/O:3	Contaminated food	Duan et al. [89]
2011	Pennsylvania	July	22	1B/O:8	Improperly pasteurized milk	Longenberger et al. [41]
2011–2014	Norway	May	21,133	2/O:9	RTE salad mix	MacDonald et al. [90,91]
2012	Tokyo	August	39	O:8	Fresh vegetable salad	Konishi et al. [92]
2013	Tokyo	April	52	O:9	Fresh vegetable salad	Konishi et al. [92]
2017–2021	France	Summer	7642	4 (87.2%); 2/3-9b (10.6%)	Barbecued sausages, chipolatas, ribs, and other pork meat products	Le Guern et al. [93]
2018–2020	Czech Republic	January	1686	-	Undercooked pork, less often vegetables, or water	Špačková et al. [94]
2019	Sweden	March–May (Spring)	57	4/O:3	Fresh spinach	Karlsson et al. [95]
2023	Austria	January-April	11	1A	Nutritional milkshakes	Colbran et al. [10]

## Data Availability

No new data were created or analyzed in this study. Data sharing is not applicable to this article.

## Abbreviations

The following abbreviations are used in this manuscript:

CFU	Colony Forming Unit
DNA	Deoxyribonucleic Acid
ECDA	European Centre for Disease Prevention and Control
EFSA	European Food Safety Authority
EU	European Union
FBO	Foodborne outbreak
HPI	High-Pathogenicity Island
IL-8	Interleukin-8
ISO	International Organization for Standardization
MAPKs	Mitogen-activated protein kinases
PCR	Polymerase Chain Reaction
PFGE	Pulsed-field gel electrophoresis
pYV	Yersinia virulence plasmid
RTE	Ready-to-eat
STEC	Shiga toxin-producing *Escherichia coli*
T3SS	Type III Secretion System
YadA	Yersinia adhesin A protein
YopA	Yersinia outer membrane protein
Yst	Yersinia stable toxin
MLST	Multilocus sequence typing
